# The estimation of the functioning of families with ASD children

**DOI:** 10.3934/publichealth.2019.4.587

**Published:** 2019-12-20

**Authors:** Anna Kostiukow, Wojciech Strzelecki, Piotr Poniewierski, Włodzimierz Samborski

**Affiliations:** 1 Department and Clinic of Rheumatology and Rehabilitation, Poznan University of Medical Sciences, 61-545 Poznań, 28 Czerwca 1956 r Street 135/147; 2 Department and Clinic of Clinical Psychology, Poznan University of Medical Sciences, Collegium Stomatologicum, Bukowska 70 Street, 60-812 Poznań

**Keywords:** autism, ASD, flexibility and cohesion evaluation scales, family functioning

## Abstract

**Background:**

Autism Spectrum Disorder (ASD) is a disease described as a neurodevelopmental disorder as the impairment of social and communication functions. Life of the people with ASD depends on the early introduction of intensive therapeutic programmes, modifying the undesirable behaviours, and aimed at teaching social and communication skills.

**Aims:**

The goal of the present work is to estimation the functioning of families with an ASD child and compare it to the functioning of families with children not diagnosed with ASD.

**Methods:**

The study was performed using Flexibility and Cohesion Evaluation Scales. The study included 70 parents of ASD children, and 70 parents with children without diagnosed ASD, as the control group.

**Results:**

The parents of children with autism achieve lower results in the Balanced Cohesion sub-scale than the control group. Also, the parents of ASD children obtained higher scores in the Disengaged sub-scale than the control group.

**Conclusions:**

The results of this papers can suggesting the risk of the appearance of a disturbed family system, functioning in families with children with ASD, which should be a trigger for providing these families with early family functioning diagnosis and consequent support and therapy.

## 1. Introduction

### 1.1. Autism

Autism Spectrum Disorder (ASD) is a disease described as strongly heterogeneous due to the large number of symptoms which may appear in the child's functioning [1], as well as the variable response of the body to the treatment process [1],[2]. In spite of the fact that the symptoms are multiple and occur with changing intensity, every person with autism presents abnormalities in communication and social interaction [2], exhibits repetitive behaviours, and a limited scope of interests [3],[4]. The onset of the disease occurs in early childhood [3]. Only a minor percentage of people with diagnosed Autism Spectrum Disorder, with mild symptoms (ex. Difficulty in social communication, problematic with adaptation to change, planning difficulty) , are able to live a relatively independent life as adults [2],[3],[5]. The majority (with symptoms of moderate and severe intensity) need the help of their families or social welfare to the end of their lives [2]. Their functioning in adult life depends on the early introduction of intensive therapeutic programmes, modifying the undesirable behaviours, and aimed at teaching social and communication skills [6]–[8].

Scientific literature stresses a constant growth in the incidence of the disorder under discussion. For example, data from the Autism and Developmental Disabilities Monitoring Network shows that, in 2012, in the USA, there were twice as many eight-year-old children with diagnosed ASD as only two years earlier, in 2010 [9]. Taking into consideration the whole population of children, in 2000, ASD was reported as occurring in one in every 150 children, and in 2010, in one of every 68 [10]. The causes of this situation are unknown. The scientists believe it is related to greater public awareness of the symptoms of autism, new diagnostic criteria, and possibility of diagnosis at a younger age [11]–[14]. These are only hypotheses, but they undoubtedly encourage various agencies-medical, social, educational and other to search for effective solutions for supporting people with autism and their families [4].

The symptoms of autism are recognised in the child's environment quite quickly. Usually, it is the parents who first realise that their child does not achieve the expected milestones in development; his or her development is retarded or stopped [15]–[17]. At that time, parents observe that their child does not react to their physical affection, does not want to express emotions, often avoids hugging (which is very hard for parents, especially for mothers) and eye contact, and does not want to communicate in any way [15],[16]. Moreover, the child may present atypical behaviours, movements related to a strong need of isolation from its surroundings, which are incomprehensible to the parents [16]. Usually, these include destructive, socially unacceptable behaviours [18]. These symptoms arouse anxiety and feelings of helplessness in the parents and make them seek professional help [15].

The problems affecting the autistic child affect also the parents. Therefore, it may be said that the autism of a child has considerable implications for its parents [19]. Caring for a child with autism is associated with emotional consequences [20]–[23]. It has been proved that parents of atypical children experience parental stress much more frequently than the general population [24], as the moment of the child's diagnosis generates strong uncertainty about the future life of the child and the whole family [25],[26].

The study by Bitsik and Sharpley, conducted on the basis of an analysis of fathers and mothers of ASD children, showed that women are more preoccupied and prone to depression than men caring for their disabled children [27]. Similar results were obtained by Dąbrowska, who indicated that mothers are much more frequently exposed to stress [28]–[30]. Moreover, it was proved that parents of ASD children are three to five times more vulnerable to depression than parents of neurotypical children [31]. The most commonly used assessment tools for preoccupation and depression [27] of parents include:

- Self-Rating Depression Scale—SDS [32].
- Self-Rating Anxiety Scale—SAS [33].
- Connor-Davidson Resilience Scale—CD-RISC [34].

### 1.2. Aim

The aim of the paper is to evaluate the functioning of families with an ASD child and compare it to the functioning of families with neurotypical children. The degree of flexibility, cohesion and level of communication enables the family to be classified either as healthy or dysfunctional.

## 2. Methods

### 2.1. Ethical approval and consent

The study was approved by Bioethics Committee of the Poznan Univeristy of Medical Sciences (approval number: 1223/17) and Australian New Zealand Clinical Trials Registry (ANZCTR) number ACTRN12618000598280.

### 2.2. Classifications and measurements

The study was performed using (*Flexibility and Cohesion Evaluation Scales*, FACES-IV) questionnaire by David H. Olson, in its Polish form, developed by Andrzej Margasiński. The questionnaire consists of sixty-two statements, to which the subject responds in a 5-degree scale, from *strongly disagree* to *strongly agree*. The statements are divided into eight sub-scales. Six of them are the main sub-scales of David H. Olson's Circumplex Model of the two dimensions of family functioning: cohesion and flexibility (Balanced Cohesion, Disengaged, Unmeshed, Balanced Flexibility, Rigid, Chaotic). The two remaining sub-scales measure family communication (which is the third dimension of the Circumplex Model) and family satisfaction. Apart from sub-scale results, it is possible to calculate three complex ratios: Cohesion Ratio, Flexibility Ratio and Total Circumplex Ratio, which reflects the degree to which family functioning is healthy [35].

#### 2.2.1. Circumplex Model

The tool used is based on the Circumplex Model, which focuses on three crucial dimensions of family functioning: cohesion, flexibility and communication. Cohesion means the emotional bonding that family members have towards one another. Flexibility of relationships is defined as the quality and expression of leadership and organization, role relationships, and relationships rules and negotiations [36]. The communication dimension is viewed as a facilitating dimension that helps families alter their levels of cohesion and flexibility. The intensity of cohesion and flexibility of family relationships may have two basic levels: balanced or unbalanced. Unbalanced cohesion may mean extremely high cohesion level (unmeshed relationships) or an extremely low cohesion level (disengaged relationships, lack of bonding). On the other hand, unbalanced flexibility may mean extremely high (chaotic family relationships) or extremely low (rigid family relationships) flexibility levels. The main hypothesis of the model says that there is a positive relationship between a balanced cohesion level, balanced flexibility level, and healthy family functioning, as well as a positive relationships between unbalanced cohesion level, unbalanced flexibility level and problematic family functioning [36].

The third basic dimension of D. H. Olson's Circumplex Model, which influences both flexibility and cohesion, is communication [37]. This refers to the skill of providing the family members with information, plans and emotions. This dimension is also defined as the positive communication skills utilized in the couple or family system[38].

Cluster analysis of data obtained from studies, using Flexibility and Cohesion Evaluation Scales, resulted in distinguishing six family types: Balanced, Cohesively Rigid, Flexibly Disengaged, Mid-range, Rigidly Disengaged and Unbalanced [35]. The Balanced type is characterised by the highest scores on the balanced sub-scales and the lowest scores on the remaining sub-scales. The Cohesively Rigid type is characterised by high scores in the balanced cohesion and rigid sub-scales, moderate enmeshed scores, and low disengaged and chaos scores. The Flexibly Disengaged type is characterised by high scores on the Balanced Flexibility and Disengaged sub-scales, and low scores on the Rigid sub-scale The Mid-range type is characterised by moderate scores on all of the sub-scales, with the exception of the disengaged sub-scale, where the score is usually low. The Rigidly Disengaged type is characterised by high scores on all of the sub-scales other than Cohesion, where moderate to low scores are characteristic. The Unbalanced type is characterised by high scores on all four of the unbalanced scales: Disengaged, Unmeshed, Rigid and Chaotic, and low scores on the two balanced scales: Balanced Cohesion and Balanced Flexibility. These families are assumed to experience the greatest difficulties and be the most problematic in terms of their functioning. It is estimated that this is the family type most often looking for therapy [35].

### 2.3. Data collection and participants

The study with Flexibility and Cohesion Evaluation Scales, by David H. Olson, in its Polish adaptation by Andrzej Margasiński, included 70 parents of ASD children, and 70 parents with children without diagnosed ASD, as the control group. The study was performed in January and February 2018. The study used inclusion criteria: (1). parents aged 25–45; (2). children without comorbidities; (3). diagnosis of autism in children.

## 3. Results

In order to compare FACES IV results obtained by the parents of ASD children and the control group, an independent samples t-test for equality of means was performed, and the statistical significance of the obtained differences was assessed.

### 3.1. Sub-scale

The analysis of the Balanced Cohesion sub-scale indicated that the parents of children with autism achieve lower FACES-IV results in the Balanced Cohesion sub-scale than the control group. The study covered 140 observations. The significance level of Levene's test indicates that the results should be interpreted with the assumed equality of variance. The p-value for the t-test for difference of means is 0.002; therefore, the means in both groups differ in a statistically significant way. The results are presented in Table 1.

**Table 1.** The sub-scales in the group of parents of children with ASD vs. parents of neurotypical children.

	Group	N	Average	P-value
Balanced Cohesion sub-scale (STEN)	Autism	70	5.2000	21,843
Control group	70	6.3571	22,135
Balanced Flexibility sub-scale (STEN)	Autism	70	5.6857	20,820
Control group	70	6.2143	19,478
Disengaged sub-scale (STEN)	Autism	70	7.2857	18,583
Control group	70	6.4143	18,217
Unmeshed sub-scale (STEN)	Autism	70	6.8857	20,610
Control group	70	5.4857	18,077
Rigid sub-scale (STEN)	Autism	70	6.9143	17,672
Control group	70	6.6857	17,573
Chaotic sub-scale (STEN)	Autism	70	6.7143	18,893
Control group	70	6.0143	19,597
Family Communication sub-scale (STEN)	Autism	70	5.3857	24,215
Control group	70	6.1714	24,846
Family Satisfaction sub-scale (STEN)	Autism	70	6.3143	24,586
Control group	70	7.2857	21,274

- **Balanced flexibility sub-scale**
  The p-value for the t-test for difference of means is 0.123; therefore, the means in both groups do not differ in a statistically significant way.
- **Disengaged sub-scale**
  The p-value for the t-test for difference of means is 0.006; therefore, the means in both groups differ in a statistically significant way.
- **Unmeshed sub-scale**
  The p-value for the t-test for difference of means is 0.000; therefore, the means in both groups differ in a statistically significant way.
- **Rigid sub-scale**
  The p-value for the t-test for difference of means is 0.444; therefore, the means in both groups do not differ in a statistically significant way.
- **Chaotic sub-scale**
  The p-value for the t-test for difference of means is 0.033; therefore, the means in both groups differ in a statistically significant way.
- **Family communication sub-scale**
  The p-value for the t-test for difference of means is 0.060; therefore, the means in both groups do not differ in a statistically significant way.
- **Family satisfaction sub-scale**
  The p-value for the t-test for difference of means is 0.014; therefore, the means in both groups differ in a statistically significant way.

The analyses within the group of parents of ASD children did not show any statistically significant differences in FACES-IV due to socio-demographic variables.

## 4. Discussion and implications

Research into parental stress levels showed that parents of children with ASD have greater uncertainty, stress and depression levels than parents of neurotypical children [39]–[43] and also parents of children with other disabilities [44],[45]. Similar results can be observed in the comparison between the stress levels of parents of ASD children and the general population [21],[43],[46]–[49].

The most significant factor generating parenting stress are the ASD symptoms in their children [31]. Among the most frequent symptoms contributing to parental stress, scientists enumerate impaired cognitive functions and impaired social reactions, which directly correspond to the emergence of parental stress, anxiety and depression [50]–[53]. Other aspects of autism which may induce parental stress are: the level of functioning of the child, the child's age, the dysfunction of adaptive behaviours, aggression, tantrums, and self-inflicted injuries [21],[54]–[57].

However, it is emphasized that there is no social understanding of the characteristics of ASD, due to which, both the parents and the ASD children themselves, are subject to more severe social criticism. The specific behaviours of ASD patients are often perceived as parenting errors [31]. What is even more important, it is considered that parental stress factors come exclusively from outside of this social group and not from the personality and behavioural models of the parents themselves [31],[58]. Important factors influencing the development of parental stress and burn-out include lack of activity of mothers of ASD children outside the home in comparison to mothers of neurotypical children, who can spend much more of their free time outside the family, in a stress-free environment [59]. Similar conclusions were made in other studies, which, apart from isolation factors, also identified the phenomenon of “self-blaming” mothers, who burden themselves with blame for their child's difficulties [60],[61]. Another aspect of parental stress, described in the literature, is escaping from the problems related to the child's disability, visible as its difficult behaviours [62].

The assessment of parental stress showed that over a half (55.8%) of fathers feel overwhelming helplessness one to five times a month. On the other hand, over 70% of mothers experience this feeling one to five times a month. The results of this study confirmed earlier research into anxiety and depression in parents, conducted by the same authors on a group of parents in Australia [27],[63].

Another study focused on the parents of children with diagnosed ASD. The research into parental stress showed that the majority of the subjects agreed to the statements that “caring for a child takes a lot of time and energy” and “the behaviour of my child embarrasses and stresses me”. In the area of social support, the majority of the subjects agreed with the statements “the members of my family rely on me” and “I cannot rely on the members of my family”. As far as the area of self-efficacy is concerned, the majority marked the answer “try another solution if the first one did not bring expected results”. The study described showed that there are multiple sources of parental stress and that its level is influenced by all members of the ADS child's family, including parents, siblings, and grandparents. It was also shown that, despite the difficulties and problems, the caregivers of ASD children have social support and can cope with difficult situations [64].

The scientific literature also includes works devoted to the role of stress resilience and self-efficacy in parents of ASD children. One of them analyses the group under discussion. The study was conducted using the Satisfaction With Life Scale (SWLS) [65], the Coping Strategy Inventory (CSI) [66], and the Coping with Stress Self-Efficacy Scale (CSSES) [67]. The results confirm that bringing up a child influences coping strategies and the sense of self-efficacy. Therefore, stress has an impact on the level of satisfaction with life of parents of ASD children. The scientists found differences depending on the parent's sex, stating that the primary goal of a woman is the sense of self-efficacy, while men put problem solving in the first place [67]–[72]. It was also shown that, together with the ageing of ASD parents, the social support for these families decreases, as does cognitive restructuring [69],[73],[74].

The results of many studies prove that the sense of self-efficacy contributes to higher life satisfaction. Moreover, the sense of self-efficacy correlates positively with resilience strategies (problem solving and cognitive restructuring) and negatively with dysfunctional strategies (social isolation, wishful thinking, self-criticism) [60],[69],[75]–[79].

It is worth mentioning the study conducted by Bitsik et al. (2017), analysing daily cortisol levels in parents of ASD children. Cortisol is called the neurohormone of stress [57],[80]. Cortisol levels were measured via the analysis of the subjects' saliva. It is estimated that cortisol is present in this material for about 10 minutes from the occurrence of the stress factor [81]. It was proved that, in 129 subjects, the levels of cortisol drop in accordance with the circadian rhythm. At the same time, the studies proved that self-inflicted injuries in children with ASD may be a stress-provoking factor in parents [57],[82].

In order to reduce parental stress, parents of ASD children are recommended to introduce effective mitigation of autism symptoms [83]. It is emphasized that only successful reduction of ASD symptoms in the child may improve the well-being of the whole family [84]. Long-term stress may have drastic health consequences on parents of ASD children [31]. Support groups for parents of ASD children are one of the forms of therapy aimed at coping with stress and preventing burn-out [85].

## 5. Conclusion

(1). It has been established that the parents of children with autism achieve lower results in the balanced cohesion sub-scale than the control group.

(2). The parents of ASD children obtained higher scores in the disengaged sub-scale than the control group.

(3). Furthermore, in the unmeshed sub-scale, their scores were higher than in the control group.

(4). In the chaotic sub-scale, the parents of ASD children obtained higher scores than the control group.

(5). It was found out that the family satisfaction level in parents of ASD children is lower than in the control group.

(6). In the balanced flexibility, rigid and family communication sub-scales, there were no statistically significant differences between the parents of ASD children and the parents from the control group.

(7). In parents of ASD children, the scores in all unbalanced sub-scales were higher than in families with children without autism (even if in some of differences were not statistically significant) while the scores in the balanced sub-scales were lower.

(8). The STEN analysis of mean results of the parents of ASD children does not show extreme results in the scales studied, their results remain in the mid-range values (with the assumption that the middle of the STEN scale is 5.5 and the standard deviation is 2).

(9). In families with ASD children, there is a higher risk of the unbalanced or rigidly disengaged family type than in families with neurotypical children.

This may be a significant result, suggesting the risk of the occurrence of a disturbed family system, functioning in families with children with ASD, which should be a trigger for providing these families with early family functioning diagnosis and consequent support and therapy.
